# Plexin-B1 silencing inhibits ovarian cancer cell migration and invasion

**DOI:** 10.1186/1471-2407-10-611

**Published:** 2010-11-08

**Authors:** Shuangmei Ye, Xing Hao, Ting Zhou, Mingfu Wu, Juncheng Wei, Yongjun Wang, Li Zhou, Xuefeng Jiang, Li Ji, Yin Chen, Lanying You, Yiqun Zhang, Gang Xu, Jianfeng Zhou, Ding Ma, Shixuan Wang

**Affiliations:** 1 Cancer Biology Research Center, Tongji Hospital, Tongji Medical College, Huazhong University of Science and Technology, Wuhan, Hubei, 430030, China; 2 Department of Gynecology, Yantai Yuhuangding Hospital, affiliated to Qingdao University Medical College, Yantai, Shandong, 264000, China

## Abstract

**Background:**

Elevated Plexin-B1 expression has been found in diverse human cancers and in non-neoplastic tissues, and it mediates diverse biological and pathological activities. However, whether or not Plexin-B1 expression is involved in human ovarian tumors remains unclear. In the present study, Plexin-B1 expression was explored in benign and malignant human ovarian tumor tissues. In addition, the impact of Plexin-B1 expression on ovarian cancer cell proliferation, migration and invasion were investigated *in vitro*.

**Methods:**

Plexin-B1 expression was analyzed in normal and benign ovarian tissues and serous ovarian tumors (both borderline and malignant) by immunohistochemical staining, as well as in four human ovarian cancer cell lines (A2780, C13*, SKOV3, and OV2008) by RT-PCR and western blot analyses. Furthermore, endogenous Plexin-B1 expression was suppressed by Plexin-B1 siRNA in SKOV3 cells, which overexpress Plexin-B1. Protein levels of Plexin-B1, AKT and AKT^Ser473 ^were examined by western blot analysis. Cell proliferation, migration and invasion were measured with MTT, wound healing and boyden chamber assays, respectively, and the cytoskeleton was monitored via F-actin staining.

**Results:**

Expression levels of Plexin-B1 protein were significantly higher in serous ovarian carcinomas than in normal ovaries or benign ovarian neoplasms, and in the former, Plexin-B1 expression was positively correlated with lymphatic metastasis, and the membrane and cytoplasm of cancer cells stained positively. SKOV3 cells displayed the highest Plexin-B1 expression at both the mRNA and protein levels among the four tested human ovarian cancer cell lines and was selected as a cell model for further *in vitro *experiments. Plexin-B1 siRNA significantly suppressed phosphorylation of AKT at Ser473 in SKOV3 cells, but it did not alter total AKT expression. In addition, silencing of Plexin-B1 in SKOV3 cells inhibited cell migration and invasion and reorganized the cytoskeleton, whereas cell proliferation was not affected.

**Conclusion:**

Plexin-B1 expression correlates with malignant phenotypes of serous ovarian tumors, probably via phosphorylation of AKT at Ser473, suggesting that Plexin-B1 might be a useful biomarker and/or a novel therapeutic target.

## Background

Plexin proteins comprise a large family of trans-membrane receptors for semaphorins, which are secreted, membrane-associated or GPI-anchored proteins originally identified for their role in axon guidance repulsion [1]. In vertebrates, nine plexins are divided into four subfamilies according to their different structures: plexinA1-4, plexinB1-3, plexin-C1 and plexin-D1 [1]. All the plexins share significant homology in their extracellular region with the scatter factor receptors c-Met and RON, whereas the highly conserved cytoplasmic region of plexins fails to show homology with any other transmembrane receptor protein [2]. Plexin-A subgroup members bind secreted class III semaphorins by associating with neuropilin-1 and neuropilin-2, and plexin-B members bind their ligands independently [3]. The majority of semaphorins are not assigned to a specific receptor [4]. To date, it is known that plexin-B1 binds Sema 4D, plexin-B2 binds Sema 4C and plexin-B3 binds Sema 5A [5].

Plexin-semaphorin interactions typically play important roles in processes such as neuron extension, axon growth guidance and the maintenance of established neuronal pathways. Recent studies have further revealed that plexins-semaphorins are also expressed outside the nervous system and that their interactions are involved in the regulation of immune responses [6-10], morphogenetic lung development [11], the cardiovascular system [12-15], the skeleton [16-18], and tumor growth and metastasis [19,20].

It is unique that Plexin-B1, a transmembrane receptor, interacts directly with Rho family GTPases through a cytoplasmic Rho GTPase binding domain (RBD) [21,22]. Rho GTPases play important roles in regulating the actin cytoskeleton during developmental processes [23] such as cell adhesion [24], cell migration, axon guidance, cell cycle events and membrane transport [25], suggesting potential involvement of Plexin-B1 in cancer progression and metastasis. Swiercz *et al*. recently reported that Plexin-B1 activates or inactivates RhoA in the presence of ErbB-2 or Met, respectively, and leads to pro- or anti-migration effects [26]. Furthermore, recent studies have shown that loss of Plexin-B1 expression in breast cancer correlates closely with ER status and indicates a more aggressive tumor phenotype [27]. In renal cell carcinomas, Plexin-B1 was down-regulated and growth of cancer cells was inhibited [28]. However, overexpression of Plexin-B1 promoted invasiveness in prostate cancer [29]. These different and even opposing results reflect the fact that the exact biological function of Plexin-B1 in tumorigenesis and cancer progression is not yet clear.

In the present study, the expression levels of Plexin-B1 protein were found to be higher in serous ovarian carcinomas than in normal ovaries or benign ovarian neoplasms. We then characterized the expression of Plexin-B1 in several ovarian cancer cell lines, and SKOV3 showed highest level of Plexin-B1. To examine the role of Plexin-B1 in SKOV3 cells, Plexin-B1 was knocked down by using three different siRNA duplexes. The results showed that silencing of Plexin-B1 inhibited migration and invasion in SKOV3 cells. Even though Plexin-B1 has been previously reported to be involved in mouse ovary follicular growth and development [4], and it might be a marker predicting unfavorable outcome in ovarian serous carcinomas [30], only a limited number of studies have investigated the role of Plexin-B1 in ovarian carcinogenesis.

## Methods

### Tissue specimens and cell lines

A collection of tissue specimens including 20 normal and benign ovarian samples (the benign ovarian tissues were histologically diagnosed as serous cystadenoma), 20 serous borderline ovarian tumors, and 80 serous ovarian cystadenocarcinomas, were obtained from the Department of Pathology of Tongji Hospital with Institutional Review Board approval. Histological type and grade were diagnosed according to the World Health Organization (WHO) criteria [31]. Staging was based on TNM classification of the International Union Against Cancer (UICC) [32]. The human epithelial ovarian adenocarcinoma cell line SKOV3 was purchased from American Type Culture Collection (ATCC, Manassas, VA, USA). The A2780 human ovarian epithelial carcinoma cell line was obtained from the European Collection of Cell Cultures (ECACC, Salisbury, UK). The OV2008 and C13* ovarian cancer cell lines were gifts from Dr. Rakesh Goel in the Ottawa Regional Cancer Center, Ottawa, Canada. SKOV3 cells were cultured in McCOY's 5A containing 10% fetal bovine serum. The other cell lines were cultured in RPMI1640 containing 10% fetal bovine serum. Cell cultures were maintained in a humidified atmosphere containing 5% CO_2 _at 37°C, and the growth medium was renewed every 2-3 days. All culture medium and supplements were purchased from Invitrogen, Carlsbad, CA, USA.

### Immunohistochemistry

Immunohistochemistry was performed on formalin-fixed, paraffin-embedded sections (5 μm thick) according to standard procedures. Briefly, after dewaxing and rehydration, antigen retrieval was done with 0.01 M citrate buffer at pH 6.0 for 15 min in a microwave oven. Then endogenous peroxidase activity was inhibited by incubating sections with 0.3% hydrogen peroxide for 30 min at 37°C, non-specific binding was blocked with normal goat serum for 30 min at 37°C, and this was followed by incubating the sections with mouse monoclonal anti-human Plexin-B1 (Santa Cruz Biotechnology, sc-28372, 1:100 dilution) at 4°C overnight. The primary antibody was detected using biotinylated anti-mouse IgG. The signal was amplified by avidin-biotin complex formation and developed with diaminobenzidine followed by counterstaining with hematoxylin. Finally, the slides were dehydrated and mounted. Plexin-B1 immunoreactivity was determined using the well-standardized H-Score system (*H = I × P*) [33]. *I *is the staining intensity, which was evaluated according to the following criteria: 0 (no staining), 1 (weak immunostaining), 2 (moderate immunostaining), 3 (strong immunostaining), and *P *is the percentage of positively stained cells in each sample. A sample with an H score ≥20 was considered Plexin-B1 positive according to a recent report [30]. Furthermore, invasive human ductal breast cancer samples that had been confirmed as highly expressing Plexin-B1 in our previous study [34] were used as positive controls, and negative controls were carried out by incubating with non-immune mouse immunoglobulin (IgG) in place of the primary antibody. Three observers who were unaware of the clinical data independently scored the sections. Discrepancies in the scoring process were reviewed, and a consensus was ultimately reached.

### RT-PCR

Total RNA was extracted from ovarian cancer cells using Trizol Reagent (Invitrogen, Life Technologies, CA, USA) following the manufacture's protocol. cDNA was synthesized from 2 μg of total RNA using Superscript reverse transcriptase (Life Technologies, CA, USA). Primer sequences used for Plexin-B1 detection were as follows, sense:5'-GCAGTGTGTTATCCTTTAATGAAA-3'; and antisense:5'-CCACTACAAACTGACCCCTC-3', producing 150-bp fragments. The housekeeping gene glyceraldehyde phosphate dehydrogenase (GAPDH) was used for normalization (sense: 5'-TGATGACATCAAGAAGGTGGTGAAG-3'; and antisense: 5'-TCCTTGGAGGCCATGTGG GCCAT-3', 240 bp product). The amplification conditions were 95°C for 1 min followed by 30 cycles of 94°C for 30 s, 58°C for 30 s, and 72°C for 45 s with a 5 min 72°C extension. PCR products were analyzed by 1.2% agarose gel electrophoresis with ethidium bromide for UV light transilluminator visualization.

### Western blotting

Confluent cells were lysed in lysis buffer (20 mM Tris-HCl, pH 7.5, 150 mM sodium chloride, 1 mM EDTA, 1 mM EGTA, 1% Triton, 2.5 mM sodium pyrophosphate, 1 mM h-glycerolphosphate, 1 mM sodium orthovanadate, 0.5 mM phenylmethylsulfonyl fluoride, and 1 mg/mL leupeptin). Protein lysates (200 μg/lane) were subjected to 8% polyacrylamide gel electrophoresis and transferred onto nitrocellulose membrane. Non-specific binding sites were blocked by incubating the nitrocellulose membrane for 1 h at 37°C with 5% nonfat dried milk in Tris-buffered saline containing 0.05% Tween-20 (TBST). Membranes were incubated overnight at 4°C with primary antibodies, then washed and incubated with horseradish peroxidase conjugated secondary antibody (Cell Signaling Technology, Danvers, MA). Bands were visualized using enhanced chemiluminescence system (ECL, Pierce, USA).

### siRNA preparation and cell transfection

Three different siRNA duplexes targeting Plexin-B1 sequences as well as a negative control siRNA were commercially designed and synthesized by Invitrogen, Carlsbad, CA, USA. The sequences were as follows: Plexin-B1 siRNA1, sense: 5'-UCAAAUUGCUGGCUACUGCAGGAGG-3'; and anti-sense: 5'-CCUCCUGCAGUAGCCAGCAAUUUGA-3'; Plexin-B1 siRNA2, sense: 5'-AACUCAGGCAAAGAGUCAGGUGCCU-3'; and anti-sense: 5'- AGGCACCUGACUCUUUGCCUGAGUU-3'; Plexin-B1 siRNA3, sense: 5'- AAGGUAUACAGACAGAUGGACAUCC-3'; and anti-sense: 5'- GGAUGUCCAUCUGUCUGUAUACCUU-3'. Negative control siRNA, sense: 5'-AGGAGAUAUUUCGAGGCUU-3'; antisense: 5'-AAGCUCGAAAUAUCUCCU-3'. The siRNA sequences were submitted to BLAST http://www.ncbi.nlm.nih.gov/blast/ to ensure their specificity of targeting.

SKOV3 cells (3 × 10^5^) were plated in 6-well plates and allowed to adhere for 24 h. 200 pmol siRNA and 5 μl Lipofectamine™ 2000 (Invitrogen) per well were diluted separately in serum-free Opti MEM (Invitrogen) for a final volume of 250 μl, gently mixed, and incubated at room temperature for 5 min. Then, the diluted siRNA solution and the diluted Lipofectamine2000 were mixed gently and incubated at room temperature for 20 min. The cells were washed with serum-free McCOY's 5A, and then the diluted siRNA/Lipofectamine2000 complex was added into the 6-well plates containing 1500 μl serum-free McCOY's 5A for 5 h, after which the complex was replaced with normal cell culture medium.

### Semi-quantitative real-time RT-PCR

Total RNA was isolated with TRIzol Reagent (Invitrogen) from cells cultured in the absence or presence of siRNA for 24 h. Real-time RT-PCR amplifications were carried out using DNase I (Promega)-treated total RNA. Reactions were performed in a Stratagene MX3000P system using Real-time PCR Master Mix (TOYOBO, Japan). Primer sequences for real-time PCR were as follows, Plexin-B1: sense, 5'-ATGTCACCTGCCAGCAGCAC-3', antisense, 5'-CAGCACTGTCCACACGCAG A-3'; 18S: sense, 5'-AGTCCCTGCCCTTTGACACA-3', antisense, 5'-GATCCGAGGGCCTCACTAAAC-3'. The reaction conditions were 95°C for 1 min followed by 40 cycles of 95°C for 15 s, 60°C for 15 s, and 72°C for 30 s. The ΔCt method was used to obtain relative expression values of Plexin-B1 as compared with 18S gene amplification.

### Cell proliferation assays

Cell proliferation was determined by 3-(4, 5-dimethylthiazol-2-yl)-2, 5-diphenyltetrazolium bromide (MTT) assay. Briefly, cells cultured in the absence or presence of Plexin-B1 siRNA or negative control siRNA for 24 h were seeded into a 96-well plate at a density of 5 × 10^3 ^per well. After being starved overnight in quiescent medium, the cells were cultured for 72 h with 10% FBS. Then, 20 μl of MTT (5 mg/ml, Sigma Chemical Co.) was added to each well and incubated for 4 h at 37°C, which was followed by adding 100 μl DMSO and incubating at 37°C for an additional 20 min. Absorbance was read at 570 nm on a microplate reader.

### Wound-healing assay

Cells were seeded in individual wells of a 6-well culture plate. Untransfected cells and cells exposed to Plexin-B1 siRNA or negative control siRNA for 5 h were cultured in quiescent medium for 24 h. Then, a sterile 10-ul pipette tip was used to longitudinally scratch a constant-diameter stripe in the confluent monolayer. The medium and cell debris were aspirated away and replaced with 2 ml of fresh serum-free medium. Photographs were taken at 0 h, 24 h and 48 h after wounding (corresponding to 24 h, 48 h and 72 h post-transfection) by phase contrast microscopy. For statistical analysis, three randomly selected points along each wound were marked, and the horizontal distance between the migrating cells and the initial wound was measured at 24 h and 48 h. Values were means ± SD from at least three-independent experiments. Differences between siRNA-treated and blank control data were determined by a Student's t-test, where *P *< 0.05 was considered significant.

### Cell invasion assay

Cell invasion was assayed using Transwell chambers (Costar, Cambridge, MA, USA) with 8-μm pore polycarbonate filters that were coated with Matrigel™ (BD Biosciences, Franklin Lakes, NJ). Cells cultured in the absence or presence of siRNA for 24 h were seeded into the upper chambers in serum-free medium at a density of 2.5 × 10^4 ^per well, and 500 μl of NIH3T3 conditioned medium was placed in the lower chamber as a chemo-attractant. After 48 h at 37°C in 5% CO_2_, the cells were fixed with 3.7% paraformaldehyde and stained with 0.1% crystal violet solution. Cells on the upper surface of the filter were removed with cotton buds. Invaded cells on the underside of the filter were photographed and counted by phase contrast microscopy (×200 magnifications). The experiments were performed in triplicate.

### Rhodamine phalloidin

SKOV3 cells were plated overnight on sterile glass coverslips in a six-well plate 24 h after a 5-h exposure to Plexin-B1 siRNA, negative control siRNA, or no treatment. The cells were washed in PBS, fixed in 3.7% paraformaldehyde, permeabilized in 0.1% Triton-X100, incubated in blocking solution (1% bovine serum albumin [BSA]), and then were kept for 20 min at room temperature in rhodamine-phalloidin solution. Images were acquired with a confocal laser scanning microscope (The Olympus FluoView FV1000).

### Statistical analysis

All experiments were repeated at least three times. The software package SPSS13.5 was used to analyze data. *P *< 0.05 was defined as statistically significant. All values were presented as means ± standard deviation (SD).

## Results

### Expression of Plexin-B1 in human ovarian tissues with different pathological changes

**Figure 1:**
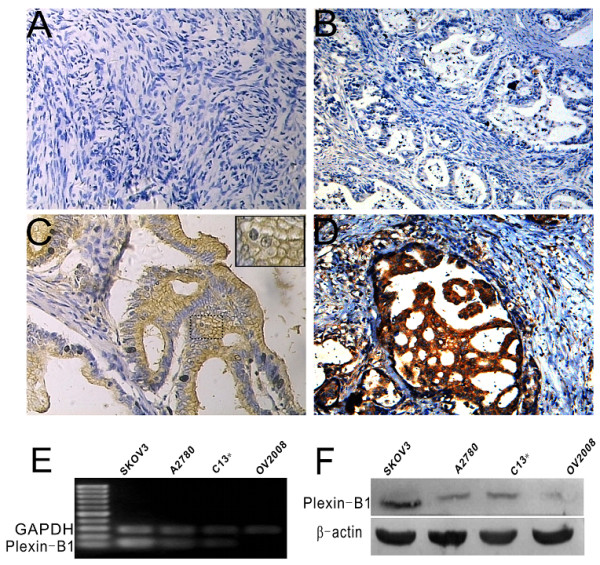
**Expression of Plexin-B1 in benign, borderline, and malignant ovarian tissues and in ovarian cancer cell lines**. Immunohistochemical staining showed the presence of Plexin-B1 in human ovarian tissues with different pathological changes. In normal ovaries (A) and benign serous ovarian cystadenomas (B), Plexin-B1 protein expression was virtually negative, whereas in serous borderline ovarian tumors, Plexin-B1 expression was higher and stained membranes; the insert shows the dashed line region at a higher magnification to demonstrate the membrane reaction product (C). In serous ovarian cystadenocarcinomas, Plexin-B1 expression was further up-regulated and showed strong cytoplasmic staining (D). Representative images are shown. Magnification, ×200. Expression of Plexin-B1 in four human ovarian cancer cell lines at both the mRNA and protein levels was detected with RT-PCR and western blotting. (E) RT-PCR showed the relative level of mRNA for Plexin-B1 in SKOV3, A2780, C13* and OV2008 cells. GAPDH was used for normalization. Product sizes: Plexin-B1, 150 bp; GAPDH, 240 bp. (F) Western blot showing Plexin-B1 protein expression in SKOV3, A2780, C13* and OV2008 cells. β-actin was used as a loading control. Product sizes: Plexin-B1, about 200 KD; β-actin, 43 KD.

To elucidate the expression of Plexin-B1 protein during serous ovarian carcinogenesis and to study the association of Plexin-B1 expression with clinical-pathological parameters, the expression levels of this protein in normal human ovaries, benign ovarian tissue, serous borderline ovarian tumors and malignant ovarian tumors were investigated with immunohistochemistry. As summarized in Table 1, Plexin-B1 immunoreactivity was present in 3 (15.00%) normal and benign ovarian samples (Figure 1A, 1B), in 7 (35.00%) borderline tumors (Figure 1C) and in 44 (55.00%) cystadenocarcinomas. The expression of Plexin-B1 in serous ovarian cystadenocarcinomas was statistically higher than that in the normal and benign tissues. In borderline tumors, the expression levels of Plexin-B1 were neither significantly higher nor significantly lower than the levels in the normal and benign tissues or the cystadenocarcinomas. Furthermore, in ovarian cystadenocarcinomas, the membrane and cytoplasm of the tumor cells was positive for Plexin-B1 immunostaining (Figure 1D), and a significant difference (*P *= 0.008) in positive staining was observed between samples with lymph node metastasis (75.00%) and those without lymph node metastasis (44.23%). Although Plexin-B1 immunoreactivity increased gradually from stage I (45.00%) to stage IV (70.00%), there were no statistical differences. No correlation was found between Plexin-B1 expression and age or cell differentiation.

**Table 1:** Expression of Plexin-B1 in ovarian tissues of different pathological changes and its correlation with clinical characteristics

		Plexin-B1 expression			
				
N = 120	Cases	Positive	Negative	Positive Percentage (%)	***P***
Normal & Benign	20	3	17	15.00	<0.001* n.s.**
Borderline	20	7	13	35.00	n.s.*
Carcinoma	80	44	36	55.00	
					
Nodal status					
Positive	28	21	7	75.00	0.008
Negative	52	23	29	44.23	
					
Tumour stage					
I	20	9	11	45.00	n.s.
II	20	10	10	50.00	
III	20	11	9	55.00	
IV	20	14	6	70.00	
					
Age(y)					
<40	32	18	14	56.25	n.s.
≥40	48	26	22	54.17	
					
Cell differentiation					
High	32	14	18	43.75	n.s.
Medium	30	18	12	60.00	
Low	18	12	6	66.67	

<0.001* and n.s.** indicated differences of Plexin-B1 expression in normal ovaries and serous ovarian cystadenomas compared with that in serous borderline ovarian tumours and serous ovarian cystadenocarcinomas respectively.

n.s.* indicated difference of Plexin-B1 expression in serous borderline ovarian tumours compared with that in serous ovarian cystadenocarcinomas.

n.s. indicated differences without statistical significance.

### Expression of Plexin-B1 in human ovarian cancer cell lines

To further investigate Plexin-B1 expression in ovarian cancer cells, RT-PCR and western blot analysis were used to detect Plexin-B1 mRNA and protein levels in four human ovarian cancer cell lines: SKOV3, A2780, C13* and OV2008. The results showed that Plexin-B1 expression levels were the highest in the SKOV3, whereas faint Plexin-B1 expression was found in the OV2008 cells (Figure 1E, F). Thus, SKOV3 cells were selected as a model for further investigation.

### Specific silencing of Plexin-B1 in SKOV3 cells with Plexin-B1 siRNA

The experiments described in this study were performed by transfection of SKOV3 cells with 200 pmol siRNA for 24 h, which offered the best silencing efficiency in our preliminary experiments.

**Figure 2:**
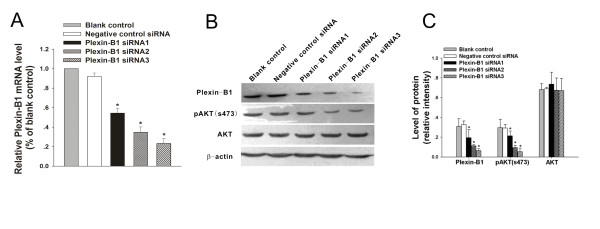
**Effects of Plexin-B1 siRNA on SKOV3 cells**. (A) Real-time PCR was performed to detect Plexin-B1 mRNA levels in non-transfected cells (Blank control) and cells at 24 h after a 5 h exposure to negative control siRNA or one of the three different Plexin-B1 siRNAs (Plexin-B1 siRNA1-3). The mean relative level ± SD of each group is shown. Plexin-B1 mRNA levels in three Plexin-B1 siRNA groups were significantly down-regulated (*P < 0.01), while the negative control siRNA did not cause an obvious change. The bar graph shows the results of three independent experiments. (B) Western blot analysis of lysates from untransfected (Blank control) SKOV3 cells and SKOV3 cells at 24 h after a 5-h exposure to one of the three different Plexin-B1 siRNAs (Plexin-B1 siRNA1-3) or to negative control siRNA. The cells were analyzed by immunoblotting with specific antibodies to Plexin-B1, p-AKT (s473), AKT and β-actin. Plexin-B1 expression was significantly down-regulated in each of the three Plexin-B1 siRNA groups relative to the blank control group or the negative control group. p-AKT (s473) expression sequentially decreased in the three Plexin-B1 siRNA groups, and AKT changed impalpably at the protein level. β-actin was the internal loading control. (C) The western blots were scanned and quantified. Data present densitometric analyses of Plexin-B1, p-AKT (s473) or AKT relative to β-actin for n = 5 independent experiments. * indicates *P *< 0.01 when compared to the blank control.

Three different siRNA duplexes targeting Plexin-B1 and a negative control siRNA were separately transferred into SKOV3 cells. At 24 h after transfection, Plexin-B1 mRNA and protein levels were assessed by real-time PCR and western blot analysis, respectively. As shown in Figure 2A-C, each of the three Plexin-B1 siRNA duplexes significantly reduced the amount of Plexin-B1 mRNA and protein. The data are from densitometric analyses of Plexin-B1: β-actin; n = 5 independent experiments.

### Silencing of Plexin-B1 down-regulats phospho-AKT (Ser473) expression in SKOV3 cells

Numerous studies showed that the PI3K/AKT pathway is constitutively overexpressed in ovarian cancers [35] and is involved in regulating malignant cellular responses such as growth, survival, migration, and invasion [36]. To investigate the relationship between Plexin-B1 expression and PI3K/AKT signaling, we monitored the effect of Plexin-B1 silencing on total AKT level and its phosphorylation level in SKOV3 cells. As shown in Figure 2B, C, inhibition of Plexin-B1 expression decreased the levels of phospho-AKT (Ser473) by more than 30%, but it did not alter total AKT expression.

### Silencing of Plexin-B1 did not affect the proliferation of SKOV3 cells

Increased AKT activity is associated with cell growth. To determine whether the Plexin-B1 siRNA-induced reduction of phospho-AKT (Ser473) would affect the proliferation of SKOV3 cells, the MTT assay was used to monitor the proliferation of untransfected SKOV3 cells and SKOV3 cells treated with Plexin-B1 siRNA2, Plexin-B1 siRNA3 or negative control siRNA. As showed in Figure 2C, there were no significant differences between the siRNA-treated groups and the untreated group (*P *> 0.05).

### Silencing of Plexin-B1 inhibited migration and invasion in SKOV3 cells

**Figure 3:**
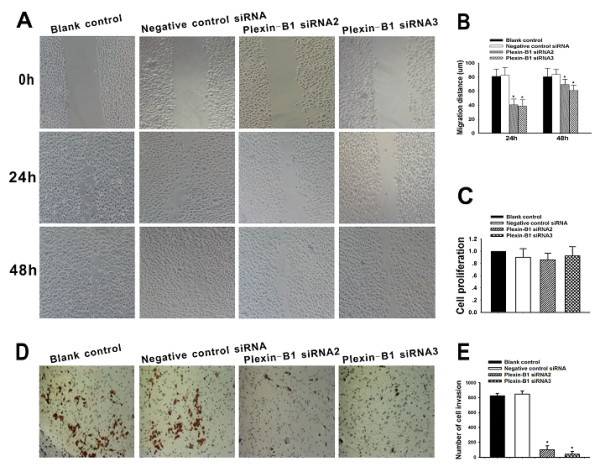
**Effect of Plexin-B1 inhibition on SKOV3 cell proliferation, migration and invasion *in vitro***. (A) Cell migration capability was determined with a wound healing assay. A confluent monolayer of untransfected SKOV3 cells or SKOV3 cells at 24 h after a 5-h exposure to Plexin-B1 siRNA2, Plexin-B1 siRNA3 or negative control siRNA (Blank control) was wounded. Photographs were taken immediately (0 h) and at 24 h and 48 h after wounding. (B) Quantification of wound closure. The data present the mean distance of cell migration to the wound area at 24 h and 48 h after wounding in three independent wound sites per group. Values are means ± SD from at least three independent experiments. (C) Analysis of the proliferation of untransfected (Blank control) SKOV3 cells and SKOV3 cells 72 h after treatment with Plexin-B1 siRNA2, Plexin-B1 siRNA3 or negative control siRNA. The data present the mean proliferation rate ± SD from three independent assays. (D) Cell invasion capability was assessed with a transwell assay. Untransfected (Blank control) SKOV3 cells and SKOV3 cells at 24 h after a 5-h exposure to Plexin-B1 siRNA2, Plexin-B1 siRNA3 or negative control siRNA were trypsinized and then plated in the upper chamber and allowed to grow for 48 h in serum-free medium. Cells that invaded the underside of the filter were fixed and stained. (D) Quantification of the transwell assay. The data present the mean number of cells on the bottom surface of the membrane from independent assays performed in triplicate. There were fewer invaded cells in the Plexin-B1 siRNA2 group (100 ± 52) and the siRNA3 group (42 ± 36) than in the blank control group (825 ± 32) or the negative control siRNA group (848 ± 41). * indicates *P *< 0.01 when compared to the blank control.

Cancer cell migration and invasion are directly related to metastasis. Plexin-B1 has been implicated in the processes of cell mobility and cancer metastasis [37,38]. To determine whether repression of Plexin-B1 expression inhibits SKOV3 cell migration and invasion, a wound-healing assay and a cell invasion assay were performed on SKOV3 cells either untransfected or transfected with Plexin-B1 siRNA2, Plexin-B1 siRNA3 or negative control siRNA for 24 h. As shown in Figure 3A-B, at 24 h and 48 h (respectively), Plexin-B1 siRNA2-treated cells showed 40% and 70% wound closure, Plexin-B1 siRNA3-treated cells showed 40% and 60% wound closure, and negative control siRNA-treated cells showed 85% and 100%, suggesting that inhibition of Plexin-B1 decreased the migration of SKOV3 cells. As shown in Figure 3D-F, the number of SKOV3 cells that passed through the filter in the Plexin-B1 siRNA2- (100 ± 52) and siRNA3- (42 ± 36) treated groups was remarkably less than that in the untreated (825 ± 32) or treated with negative control siRNA (848 ± 41) groups, which shows that inhibition of Plexin-B1 expression suppressed SKOV3 cell invasion *in vitro*.

### Silencing of Plexin-B1 altered the cytoskeleton of SKOV3 cells

**Figure 4:**
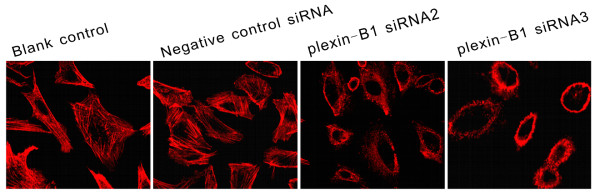
**Effect of Plexin-B1 inhibition on cytoskeleton rearrangement**. Representative cytoskeletal rearrangements (phalloidin-fluorescein isothiocyanate staining) of untransfected (Blank control) SKOV3 cells and SKOV3 cells at 24 h after a 5-h exposure to Plexin-B1 siRNA2, Plexin-B1 siRNA3 or negative control siRNA. Repression of Plexin-B1 by Plexin-B1 siRNA2 and siRNA3 inhibited F-actin polymerization and filopodia formation in SKOV3 cells. Red indicates F-actin.

A previous study proved that Plexin-B1 regulates the actin cytoskeleton by activating Rho activity [22]. To investigate how changes in Plexin-B1 expression affect the reorganization of the cytoskeleton, rhodamine-phalloidin was used to label F-actin in SKOV3 cells 24 h after a 5-h exposure to Plexin-B1 siRNA2 or siRNA3. As shown in Figure 4, SKOV3 cells in the transfected groups shrank without F-actin polymerization or filopodia formation, whereas cells in the blank and control groups showed pointed filopodia or polygonal cell shapes with pointed edges, in which brightly stained longitudinal actin bundles were detected [39].

## Discussion

Plexins are a family of transmembrane receptors that mediate repulsive neural responses induced by semaphorins. Increasing evidence suggests that plexins have functions in multiple types of tumors and multiple developmental processes, such as cell adhesion, migration, and apoptosis. Plexin-B1, a high-affinity receptor for Sema 4D, is widely expressed in tissues outside the nervous system and regulates angiogenesis [37,40], oncogenesis [27], cell mobility [26,41], mouse ovary follicular growth and hormonogenesis [4] by initiating different signaling pathways via its cytoplasmic region, which binds and activates various intracellular molecules. It has been reported that both the expression and roles of Plexin-B1 are closely associated with cellular types and with intracellular signaling molecules such as the receptor tyrosine kinases ErbB2 and MET [26]. However, the biological effects of Plexin-B1 are poorly understood.

*Valente et al*. [30] recently assessed the potential value of Plexin-B1 expression, either alone or in conjunction with Met, as a marker of tumor progression by analyzing their expression in a series of 50 malignant epithelial tumors (35 breast and 15 ovary). Plexin-B1 expression was found in 7/11 ovarian serous adenocarcinomas, and 6/15 ovarian adenocarcinomas expressed both Met and Plexin-B1, which was predictive of an unfavorable outcome. In the present study, immunohistochemical analysis was used to investigate Plexin-B1 expression in ovarian serous tumors in a larger series. Consistent with data reported previously by *Valente et al*., our results revealed that elevated expression of Plexin-B1 was associated with increasing tumor malignancy. Plexin-B1 levels ranged from 15.00% in normal and benign ovarian tissues through 35.00% in borderline tumors to 55.00% in malignant ovarian tumors, suggesting that Plexin-B1 might be an early factor in ovarian serous tumor development. Furthermore, the tumors with lymph node metastasis expressed higher levels of Plexin-B1 than those without lymph node metastasis. Indeed, Plexin-B1 expression in precursor lesions suggested a certain role of this protein in early ovarian carcinogenesis before invasion or metastasis. It has been reported that Sema 4D might exert its proangiogenic effect via Plexin-B1 to promote tumor growth and survival and thereby facilitate tumor metastasis in head and neck squamous cell carcinomas and other epithelial-derived tumors [37]. However, Plexin-B1 protein may have additional roles in different types of tumors. For example, Plexin-B1 is absent in more than 80% of renal cell carcinomas but present in all kinds of renal tubules [28]. A possible mechanism underlying this expression status is that the Plexin-B1 gene is located in 3p21, which is a frequently deleted region in renal cell carcinomas.

Recent studies have provided evidence that, in response to Sema 4D, Plexin-B1 stimulates endothelial cell migration by activating the PI3K/AKT signaling pathway [3,41]. The present work showed that reducing Plexin-B1 expression in SKOV3 cells inhibited AKT activity by reducing the level of phospho-AKT (Ser473), which plays important roles in regulating cell proliferation, migration and invasion. However, the proliferation rate of SKOV3 cells was not affected by Plexin-B1 knock-down, suggesting that Plexin-B1 has no essential role in regulating the proliferation of SKOV3 cells. Swiercz *et al*. [26] demonstrated that Plexin-B1 activates the the PI-3-kinase/Akt pathway in response to Sema 4D, but requires ErbB-2 to activate RhoA and consequently promote cell migration. Interestingly, they also suggested that Plexin-B1 inhibits RhoA activity in the presence of Met, resulting in an antimigratory effect. They proposed a novel mechanism by which Plexin-B1-mediated signaling can be regulated via differential association with ErbB-2 and Met, and the cellular effects elicited by Plexin-B1 are hypothesized to be determined by the relative expression levels of ErbB-2 and Met, which compete to mediate Sema 4D's effects. In our study, the SKOV3 cells expressed both Met and ErbB-2 [42,43], and inhibition of Plexin-B1 resulted in reductions in cell motility and invasive capacity. These results indicate that Plexin-B1 may principally interact with ErbB-2 to activate AKT in SKOV3 cells and consequently stimulate the PI3K/AKT pathway, which plays a key role in the progression and metastasis of various human ovarian cancers [35].

To date, Plexin-B1 has been shown to induce the reorganization of the cytoskeleton by stimulating different downstream adaptors. On the one hand, dimerization of Plexin-B1 is sufficient to induce ErbB2-dependent activation of Rho and to regulate the remodeling of the actin cytoskeleton and alter cell movement in response to Sema 4D [22]. On the other hand, it has been shown that Plexin-B1 inactivates PI3K, dephosphorylates AKT and activates GSK-3β through R-Ras GAP activity. Activation of GSK-3β leads to inhibition of microtubule polymerization and stabilization [44]. In this study, we found that effective suppression of Plexin-B1 in SKOV3 cells led to cell shrinkage and loss of polarity without F-actin polymerization or filopodia formation. The loss of cell polarity in terms of actin assembly and stable microtubule distribution suggested that there should also be defects in cell migration and invasion [45]. Furthermore, several other regulators of the actin cytoskeleton may also be implicated in the Plexin-B1-induced reorganization of SKOV3 cells. For example, the MEK/ERK signaling pathway, which was reported to be constitutively activated in epithelial ovarian cancer cells [46], has been shown to restore stress fibers in transformed fibroblasts [47], and a recent study suggested that Plexin-B1 can utilize RhoA to stimulate ERK [48].

However, in this report, it remains uncertain whether the reduced AKT activity induced by Plexin-B1 inhibition and the corresponding cellular responses are the result of Sema 4D produced by SKOV3 cells acting on the Plexin-B1 receptor to stimulate downstream molecules via an autocrine/paracrine mechanism, or whether overexpression and consequent clustering of Plexin-B1 in SKOV3 cells leads to phosphorylation of its cytoplasmic region. We have produced a Sema 4D fusion protein *in vitro *and further investigations are under way. In addition, studies addressing whether this oncogenic function of Plexin-B1 in SKOV3 cells is a cell type-specific effect or instead is shared across multiple ovarian cancer cell lines are ongoing.

## Conclusions

Plexin-B1 was strongly expressed in tissues of patients with ovarian serous adenocarcinomas and its expression was found to be associated with lymph node metastasis. *In vitro *assays indicated that Plexin-B1 contributes to tumor migration and invasion. Additional studies are warranted to elucidate the role of Plexin-B1 as a possible tumor marker as well as a putative therapeutic target for ovarian serous tumors.

## Abbreviations

siRNA: short interference RNA; AKT^Ser-473^: AKT at serine 473; Sema 4D: semaphorin 4D.

## Competing interests

The authors declare that they have no competing interests.

## Authors' contributions

SY carried out the immunohistological staining, participated in functional assays and drafted the manuscript. XH carried out SKOV3 cell proliferation changes upon Plexin-B1 silencing; performed assays to detect the effect of Plexin-B1 siRNA2 on the migration, invasion and cytoskeleton of SKOV3 cells; and revised the manuscript. TZ, MW and JW designed the study. YW and LZ guided and helped SY in the immunohistochemical staining and analyzed the immunohistochemical data. XJ and LJ supported the molecular studies and data analysis. YC and LY participated in the design of the study and provided expertise in molecular biological techniques. YZ contributed the statistical data analysis. GX and JZ participated in the design and coordination of the study. DM and SW participated in the data interpretation and critically revised the Manuscript. All the authors have read and approved the final manuscript.

## Pre-publication history

The pre-publication history for this paper can be accessed here:

http://www.biomedcentral.com/1471-2407/10/611/prepub
