# Correction to: Plexin-B1 silencing inhibits ovarian cancer cell migration and invasion

**DOI:** 10.1186/s12885-019-6367-9

**Published:** 2019-11-29

**Authors:** Shuangmei Ye, Xing Hao, Ting Zhou, Mingfu Wu, Juncheng Wei, Yongjun Wang, Li Zhou, Xuefeng Jiang, Li Ji, Yin Chen, Lanying You, Yiqun Zhang, Gang Xu, Jianfeng Zhou, Ding Ma, Shixuan Wang

**Affiliations:** 1 Cancer Biology Research Center, Tongji Hospital, Tongji Medical College, Huazhong University of Science and Technology, Wuhan, 430030 Hubei China; 2 Department of Gynecology, Yantai Yuhuangding Hospital, affiliated to Qingdao University Medical College, Yantai, 264000 Shandong China

**Correction to: BMC Cancer (2010) 10:611**

**https://doi.org/10.1186/1471-2407-10-611**

Following publication of the original article [1], the authors reported the following error is the article:

The image of Blank control 48 h in the lower left of Fig. 3a was in fact a photograph coming from another group [Blank control 24 h group shown in the middle left of Fig. 3a], they rearranged and checked throughout the original data, statistical analysis of the summarized results was shown in Fig. 3b, c and e. The corrected figure is presented below.

**Fig. 3:**
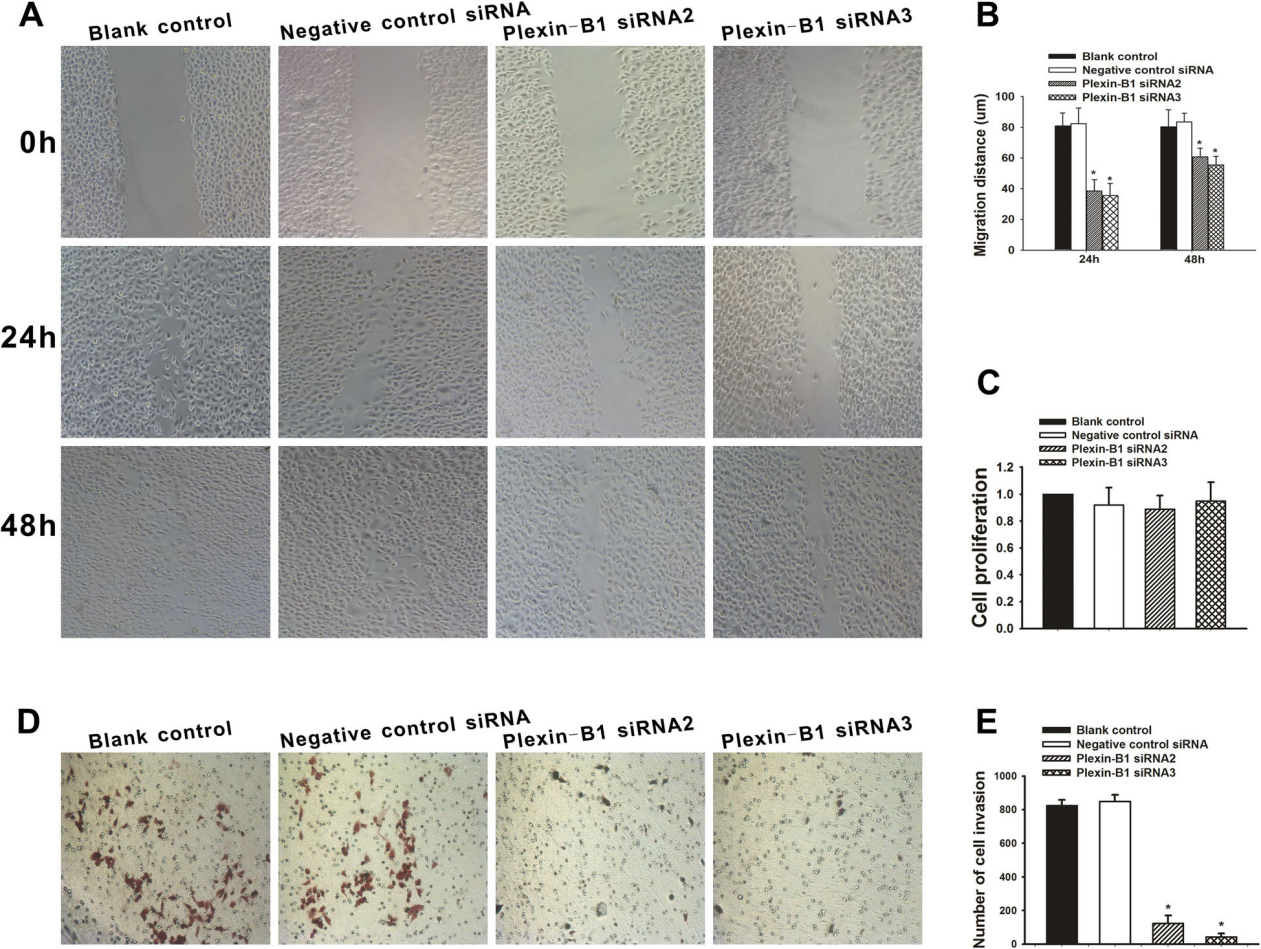
Effect of Plexin-B1 inhibition on SKOV3 cell proliferation, migration and invasion in vitro. **a** Cell migration capability was determined with a wound healing assay. A confluent monolayer of untransfected SKOV3 cells or SKOV3 cells at 24 h after a 5-h exposure to Plexin-B1 siRNA2, Plexin-B1 siRNA3 or negative control siRNA (Blank control) was wounded. Photographs were taken immediately (0 h) and at 24 h and 48 h after wounding. **b** Quantification of wound closure. The data present the mean distance of cell migration to the wound area at 24 h and 48 h after wounding in three independent wound sites per group. Values are means ± SD from at least three independent experiments. **c** Analysis of the proliferation of untransfected (Blank control) SKOV3 cells and SKOV3 cells 72 h after treatment with Plexin-B1 siRNA2, Plexin-B1 siRNA3 or negative control siRNA. The data present the mean proliferation rate ± SD from three independent assays. **d** Cell invasion capability was assessed with a transwell assay. Untransfected (Blank control) SKOV3 cells and SKOV3 cells at 24 h after a 5-h exposure to Plexin-B1 siRNA2, Plexin-B1 siRNA3 or negative control siRNA were trypsinized and then plated in the upper chamber and allowed to grow for 48 h in serum-free medium. Cells that invaded the underside of the filter were fixed and stained. **d** Quantification of the transwell assay. The data present the mean number of cells on the bottom surface of the membrane from independent assays performed in triplicate. There were fewer invaded cells in the Plexin-B1 siRNA2 group (100 ± 52) and the siRNA3 group (42 ± 36) than in the blank control group (825 ± 32) or the negative control siRNA group (848 ± 41). * indicates *P* < 0.01 when compared to the blank control
